# Sucralose Stimulates Mitochondrial Bioenergetics in Caco-2 Cells

**DOI:** 10.3389/fnut.2020.585484

**Published:** 2021-01-18

**Authors:** Juan Carlos Bórquez, Miltha Hidalgo, Juan M. Rodríguez, Alejandra Montaña, Omar Porras, Rodrigo Troncoso, Roberto Bravo-Sagua

**Affiliations:** ^1^Laboratorio de Investigación en Nutrición y Actividad Física, Instituto de Nutrición y Tecnología de los Alimentos, Universidad de Chile, Santiago, Chile; ^2^Laboratory for Research in Functional Nutrition, INTA, Universidad de Chile, Santiago, Chile; ^3^Advanced Center for Chronic Diseases, Universidad de Chile, Santiago, Chile; ^4^Laboratory of Obesity and Metabolism in Geriatrics and Adults, INTA, Universidad de Chile, Santiago, Chile; ^5^Chile State Universities Network on Aging, Universidad de Chile, Santiago, Chile

**Keywords:** sucralose, metabolism, mitochondria, Ca^2+^, artificial sweetener

## Abstract

Sucralose is a non-caloric artificial sweetener widely used in processed foods that reportedly affects energy homeostasis through partially understood mechanisms. Mitochondria are organelles fundamental for cellular bioenergetics that are closely related to the development of metabolic diseases. Here, we addressed whether sucralose alters mitochondrial bioenergetics in the enterocyte cell line Caco-2. Sucralose exposure (0.5–50 mM for 3–24 h) increased cellular reductive power assessed through MTT assay, suggesting enhanced bioenergetics. Low doses of sucralose (0.5 and 5 mM) for 3 h stimulated mitochondrial respiration, measured through oxygraphy, and elevated mitochondrial transmembrane potential and cytoplasmic Ca^2+^, evaluated by fluorescence microscopy. Contrary to other cell types, the increase in mitochondrial respiration was insensitive to inhibition of mitochondrial Ca^2+^ uptake. These findings suggest that sucralose alters enterocyte energy homeostasis, contributing to its effects on organismal metabolism.

## Introduction

Sucralose belongs to the group of non-caloric artificial sweeteners (NAS or NNS), is synthesized by the selective halogenation of sucrose, being 600 times sweeter than sucrose (1). The U.S. Food and Drug Administration approved its use in humans in 1998. Strikingly, NAS consumption in the U.S. increased by 200% in children and 54% in adults from 1999 to 2012 (2). Recently, its metabolic effects are gaining scientific attention. Sucralose reportedly induces glucose intolerance and decreases insulin sensitivity in mice and healthy subjects (3–6). In this sense, sucralose exposure also increases glucose-induced insulin secretion in MIN6 β pancreatic cell line (7). In opposition, studies shown no effects of sucralose on glucose homeostasis in healthy humans (8–10). At the intestinal level, sucralose affects the composition of the gut microbiota (11), disrupts the intestinal epithelial barrier (12), and exacerbated intestinal inflammatory reactivity in an animal model of Crohn's disease (13). Interestingly, it has been reported that sucralose consumption may impact energy homeostasis independently gut microbiota (9, 14). Therefore, its metabolic effects remain unclear and the mechanisms involved are only partially understood.

In this regard, mitochondria are critical regulators of energy homeostasis, and their dysfunction is associated with metabolic diseases (15). Their primary function is to generate adenosine triphosphate (ATP) through oxidative phosphorylation (OXPHOS). Nevertheless, mitochondria also maintain glucose homeostasis, response to stress, inflammation, redox balance, and cell death (16). Diverse signals regulate mitochondrial bioenergetics, and among them, intracellular calcium (Ca^2+^) is one of the most prominent. Ca^2+^ readily enters into the mitochondrial matrix, driven by the mitochondrial transmembrane potential, through the Mitochondrial Ca^2+^ uniporter 1 (MCU1) channel (17). While a moderate increase in mitochondrial Ca^2+^ boosts mitochondrial oxidative function, an excessive and prolonged increase induces apoptotic cell death (16).

Interestingly, studies showed that sucralose increases Ca^2+^ in MIN6 cells (7). Therefore, sucralose may impact mitochondrial metabolism, but this has not been investigated. Remarkably, Roberts et al. showed that a single oral dose of ^14^C-sucralose (1 mg/kg) in eight male subjects had a mean absorption of 14.5% (range 8.9–21.8%) and a mean excretion of 78.3% (range 69.4–89.6%) in feces (1). Since most of the sucralose remains in the gastrointestinal tract during digestion, this suggests that the gastrointestinal cells, such as enterocytes, have the highest exposure to sucralose. Thus, we aimed to evaluate the impact of sucralose on mitochondrial bioenergetics in the enterocyte human cell line Caco-2. We show that sucralose increases mitochondrial bioenergetics by increasing mitochondrial transmembrane potential and respiration, cytosolic Ca^2+^ mobilization, without changes in cellular ATP levels. Those changes in mitochondria are independent of mitochondrial Ca^2+^ entry.

## Methods

### Cells Culture

Caco-2 (HTB-37TM) cells were acquired from the American Type Culture Collection (ATCC®, Manassas, VA). We used Caco-2 cells of a high passage number (p.95-105) maintained at 37°C in a humidified incubator in 5/95% CO_2_/air. Growth medium consisted of high-glucose Dulbecco's Modified Eagles Medium (DMEM, 11995-040, Thermo Fisher Scientific, Waltham, MA) with 10% (v/v) FBS (04-001-1A-US, Biological Industries, Beit HaEmek, Israel), 1 mM sodium pyruvate, 4 mM L glutamine, 100 U/ml penicillin and 100 mg/ml streptomycin. The medium was replaced on alternate days.

### Sucralose

0.25 M sucralose (69293, Sigma-Aldrich, St. Louis, MO) stock solution was prepared in DMEM. After filtration through a 0.22 μm-pore membrane, the stock solution was diluted with complete medium to yield the final concentrations (adjusted to pH 7.4) for the experiments.

### 3-[4,5-Dimethylthiazol-2-yl]-2,5-Diphenyltetrazolium Bromide (MTT) Cell Viability Assay

Cells were seeded in 96-wells culture plates (40,000 cells/well) and grown for 2 days to 90% confluence. Then, cells were exposed to 0, 0.5, 1, 5, 15, or 50 mM sucralose for 3, 6, 18, or 24 h. Then, 100 μL of 5 mg/mL MTT solution (M5655, Sigma-Aldrich) was added to each well and incubated at 37°C for 1 h. After the MTT reduction to formazan, crystals were dissolved in isopropanol. Absorbance was read at 570 nm with a reference wavelength of 655 nm. One percentage (v/v) Triton X-100 treated was used as a control of cell death. Measurements were performed in triplicate (technical replicates). Results are expressed as the percentage of control.

### Oxygen Consumption Assessment

Cells were seeded in 60 mm culture plates (3 × 10^6^ cells/plate) and grown for 2 days until reaching 90% confluence. Then, cells were exposed to 0 (control), 0.5 or 5 mM sucralose for 3 h. Then cells were trypsinized, pelleted, and resuspended in PBS. The suspension was placed in a chamber coupled to Clark's electrode (Oxygraph, Hansatech Instruments, Norfolk, UK). Oxygen levels were recorded for 3 min intervals at baseline, non-ATP-associated (400 nM oligomycin, 75351, Sigma-Aldrich), and uncoupled (12 μM CCCP, C2759, Sigma-Aldrich) conditions. For experiments in the presence of 10 μM ruthenium red (RuRed, R2751, Sigma-Aldrich), RuRed was added 10 min before sucralose treatment. Experiments were performed without technical replicate. Oxygen consumption rates were calculated and normalized for protein content by Novagen BSA assay (71285-3, Sigma-Aldrich). Results are shown as fold change with respect to the control condition.

### ATP Measurement

Cells were seeded in 96-wells culture plates (40,000 cells/well) and grown for 2 days until reaching 90% confluence. Then, cells were exposed to 0, 0.5, 1, 5, 15, or 50 mM sucralose for 3, 6, 18, or 24 h. Intracellular ATP levels were determined with a luciferin/luciferase-based ATP detection kit (TB288, Promega, Madison, WI) according to manufacturer instructions. Sample luminescence was quantified in a TopCount NXT microplate luminescence counter (PerkinElmer, Waltham, MA). Five microgram per milliliter oligomycin treatment for 3 h was used as a control to deplete mitochondria-derived ATP. Measurements were performed in quintuplicate (technical replicates). Data were normalized and expressed as fold change with respect to the control condition.

### Western Blot

Cells were seeded in 60 mm culture plates (3 × 10^6^ cells/plate) and grown for 2 days until reaching 90% confluence. Then, cells were exposed to 0 (control), 0.5 or 5 mM sucralose for 3 h. Then, cells were scraped and lysed with T-PER lysis buffer (78510, Thermo Fisher Scientific) supplemented with commercial phosphatases and protease inhibitors (04906845001 and 11836153001, Roche, Basel, Switzerland) and centrifuged at 1,000 rcf for 10 min at 4°C. The supernatant protein content was assessed using the Novagen BCA assay and denatured in SDS buffer. Thirty microgram of proteins were separated by SDS–PAGE (10% polyacrylamide), electrotransferred onto PVDF membranes, and blocked with 5% nonfat milk in 0.1% v/v Tween 20 Tris-buffered saline (pH 7.6). Membranes were incubated in 0.1% v/v Tween 20 Tris-buffered saline with 1:1,000 primary antibodies against Thr172 p-AMPKα (2535, Cell Signaling Technology Danvers, MA), AMPKα (2532, Cell Signaling Technology) or GAPDH (14C10, Cell Signaling Technology) at 4°C overnight and re-blotted with horseradish peroxidase-linked secondary antibody (1:5,000). Finally, the bioluminescent signals were detected using ECL-plus reagent (DW1029, Biological Industries) and C-DiGit Blot Scanner (LI-COR, Lincoln, NE). Bands were analyzed with Image J version 1.51 software (National Institutes of Health, Bethesda, MD). Experiments were performed without technical replicate. Protein content was normalized by GAPDH level and data were expressed as fold change with respect to control condition.

### Cytosolic Ca^2+^ Levels and Mitochondrial Transmembrane Potential Imaging

Cytosolic Ca^2+^ levels and mitochondrial transmembrane potential were monitored by live-cell epifluorescence microscopy using Fura-2 AM (F1221, Thermo Fisher Scientific) and Tetramethylrhodamine Methyl Ester (TMRM, I34361, Thermo Fisher Scientific) dyes, respectively. Cells were seeded on glass coverslips (Marienfeld, Germany) in 35 mm culture plates (0.1 × 10^6^ cells/plate) and grown for 2 days until reaching 25% confluence. Then, coverslips were placed on an open recording chamber with Krebs Ringer HEPES (KRH) buffer (in mM: 140 NaCl, 4.7 KCl, 20 HEPES, 1.25 MgSO4, 1.25 CaCl2; pH 7.4) supplemented with 5 mM glucose (KRH-GLC). Dyes were prepared in 0.01% pluronic acid DMSO. Cells were loaded for 30 min at room temperature with either 5 μM Fura-2 AM or 400 nM TMRM in KRH-GLC. After that time, dye excess was removed by rinsing cells three times with KRH-GLC. Imaging took place in an inverted microscope (Nikon Ti, Tokyo, Japan) equipped with a 40x NA 1.3 oil objective. Dual excitation at 340 and 380 nm for Fura-2 AM and 548 nm for TMRM was achieved with a xenon lamp coupled to a monochromator device (Cairn Research Ltd., Faversham, UK). Emission over 520 and 574 nm for Fura-2 AM and TMRM, respectively, was collected using long-pass filters and digitalized by a cooled charge-coupled device camera (Hamamatsu ORCA 03, Hamamatsu, Japan) at a 1 frame/s rate. For Fura-2 AM, baseline fluorescence was measured for 25 min and then for 2 h after the addition of KRH-GLC (Control), 0.5 mM, or 5 mM of sucralose. For TMRM experiments, cells were pre-treated with KRH-GLC (control), 0.5 or 5 mM of sucralose for 3 h, and then imaged for 25 s at 1. The resulting set of TMRM images was averaged to obtain end-point fluorescence. Experiments were performed without technical replicate. Image analysis was performed using the Micromanager software (San Francisco, CA, USA). Four to seven cells were analyzed and averaged per independent sample. Data are expressed as fold change in the 340/380 nm fluorescence ratio compared to baseline, in the case of Fura-2 AM, and arbitrary units in TMRM.

### Statistical Analysis

Results are shown as means ± SEM. Greater than or equal to four independent experiments were performed to proceed with statistical tests (biological replicates). Data were analyzed using one-way ANOVA followed by a Holm-Sidak post-test. *P* < 0.05 was considered statistically significant.

## Results

### Sucralose Stimulates Mitochondrial Bioenergetics in Caco-2 Cells

**Figure 1:**
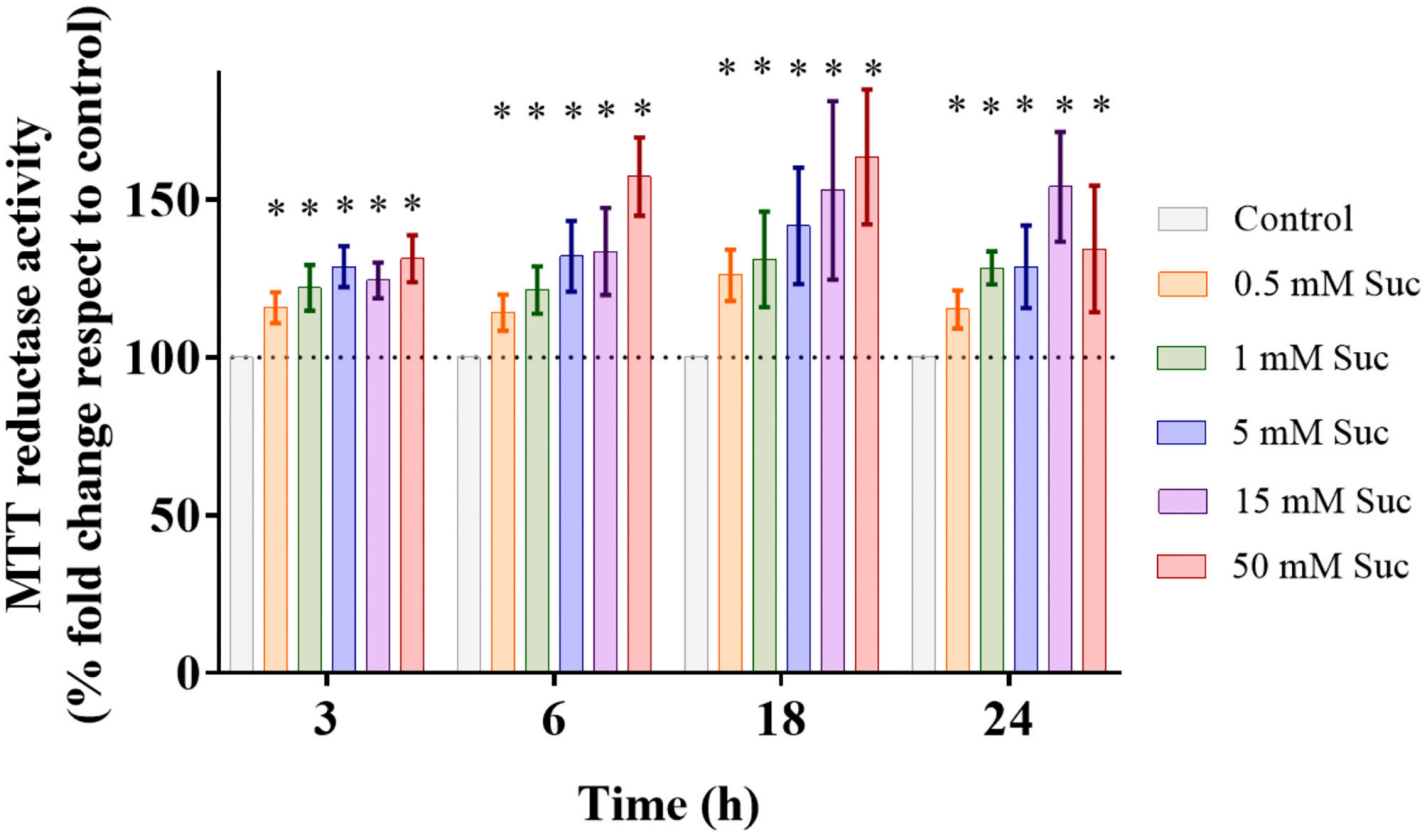
Sucralose increases cellular reductive power. Caco-2 cells (p.95-105) were cultured in control condition or treated with 0.5, 1, 5, 15, or 50 mM sucralose (Suc) for 3, 6, 18, and 24 h Then, their MTT reductase activity was measured through MTT assay (*n* = 4–5). Data are shown as mean ± SEM. *P < 0.05 compared with control cells of the respective time using one-way ANOVA followed by a Holm-Sidak post-test.

First, we evaluated whether sucralose administration affects the Caco-2 cell viability by performing the MTT assay. In all experimental conditions, from 0.5 to 50 mM for 3–24 h, we observed significant increases in the cellular reducing capacity that were dose- and time-dependent (Figure 1). This technique is based on the intracellular reduction of MTT to formazan, a redox reaction that occurs mainly at mitochondria. These results suggest that sucralose increases mitochondrial reductive power. Van Eyk et al. showed that doses of artificial sweeteners >10 mM *in vitro* generate alterations in cell morphology, attributable to high osmolarity effects (18). Therefore, we decided to use 0.5 and 5 mM sucralose concentrations, and a 3 h exposure time to study short-term downstream cell signaling and mitochondrial bioenergetics.

**Figure 2:**
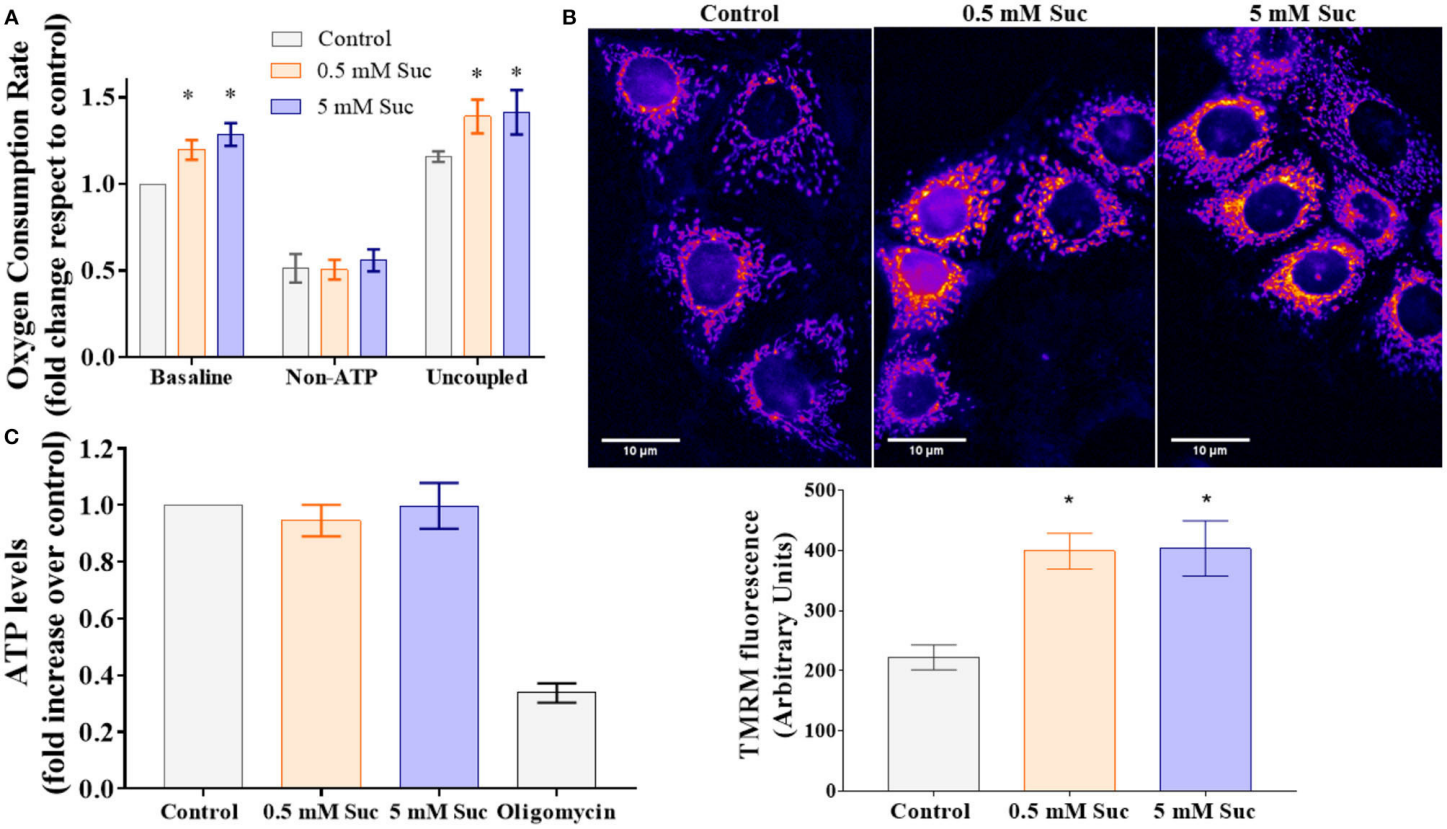
Sucralose stimulates mitochondrial bioenergetics in Caco-2 cells. **(A)** Sucralose increases cell respiration. Caco-2 cells (p.95-105) were cultured in control condition or treated with 0.5 or 5 mM sucralose (Suc) for 3 h. Their oxygen consumption rate was measured through oxygraphy using Clark's electrode (*n* = 4–5). Baseline, non-ATP-associated (400 nM oligomycin) and uncoupled (12 μM CCCP) respiration conditions were analyzed. *P < 0.05 compared with control respiration in the same condition. **(B)** Sucralose increases mitochondrial transmembrane potential. Caco 2 cells (p95-105) were cultured in control condition or treated for 3 h with 0.5 or 5 mM sucralose. Then, their mitochondrial transmembrane potential was measured as the incorporation of the voltage-sensitive fluorescent mitochondrial dye TMRM by live-cell epifluorescence microscopy. Upper panel: Representative images of TMRM-stained Caco-2. Lower panel: Quantification of TMRM fluorescence, arbitrary units (*n* = 4). *P < 0.05 compared with control cells. **(C)** Sucralose does not change ATP levels. Caco-2 cells (p95-105) were cultured in control condition or treated with 0.5 or 5 mM sucralose for 3 h. ATP levels were measured through luciferin/luciferase-based ATP assay (*n* = 5–6). All data are shown as mean ± SEM and were analyzed using one-way ANOVA followed by a Holm-Sidak post-test.

To assess mitochondrial bioenergetics, we measured respiration as the oxygen consumption rate associated with OXPHOS. We observed that both 0.5 and 5 mM sucralose increased baseline and uncoupled respiration compared to control (Figure 2A). We did not find differences between both sucralose concentrations. Sucralose exposure did not change non-ATP-associated respiration, suggesting that this sweetener did not affect extra-mitochondrial oxygen consumption. Altogether, these results indicate that sucralose boosts mitochondrial oxidative metabolism.

Mitochondrial transmembrane potential generation relies on mitochondrial respiration, which leads to proton translocation through the mitochondrial inner membrane. This process shapes one of the most distinctive aspects of functional mitochondria, its high negative electrochemical potential at the mitochondrial matrix. We measured mitochondrial transmembrane potential with the cationic dye TMRM in living cells in the control condition or treated with sucralose. Fluorescence images showed an intracellular fluorescence with a distribution consistent with a mitochondrial pattern (Figure 2B, upper panel). After 3 h of exposure, both 0.5 and 5 mM sucralose increased TMRM fluorescence compared to control, which suggests that sucralose increases mitochondrial transmembrane potential in Caco-2 cells (Figure 2B, lower panel).

**Figure 3:**
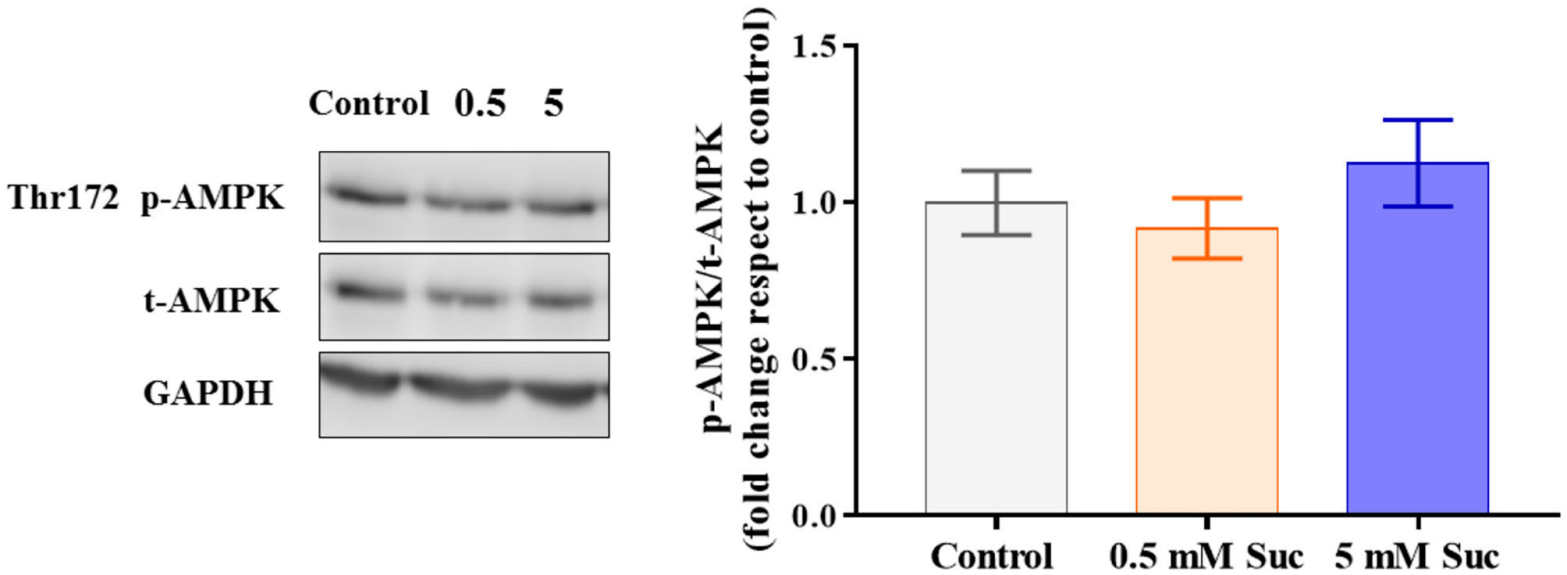
Sucralose did not change AMPK protein levels in Caco-2 cells. Caco-2 cells (p.95-105) were cultured in control condition or treated with 0.5 or 5 mM sucralose (Suc) for 3 h. Thr172 p-AMPK, total AMPK and GAPDH protein levels were determined through western blot (*n* = 6). Data are shown as means ± SEM (*n* = 6) and groups were compared using one-way ANOVA followed by a Holm-Sidak post-test.

The mitochondrial transmembrane potential is the driving force for ATP synthesis by the mitochondrial ATP synthase. Thus, we measured the ATP levels in response to treatment with sucralose, which did not show differences compared to control (Figure 2C). Also, we measured AMPK activation as a potential indicator of cellular metabolic stress. Consistent with the unchanged ATP levels, we did not observe AMPK phosphorylation changes in response to sucralose (Figure 3). These results suggest that sucralose does not acutely change the cellular energy state.

### Sucralose Increases Mitochondrial Bioenergetics in Caco-2 Cells Independently of Mitochondrial Ca2^+^ Entry

**Figure 4:**
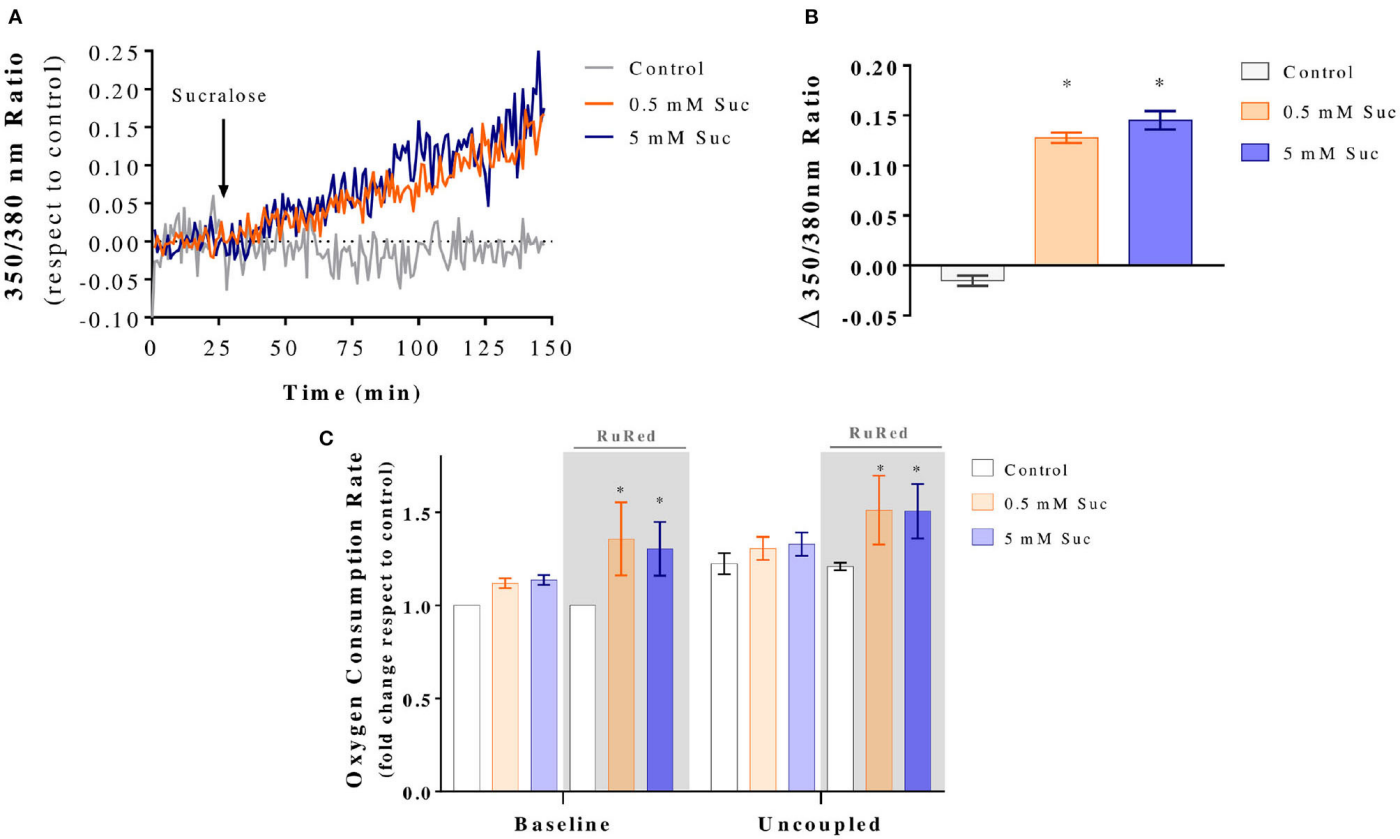
Sucralose increases mitochondrial bioenergetics in Caco-2 cells independently of mitochondrial Ca^2+^ entry. **(A)** Sucralose acutely increases cytosolic Ca^2+^. Caco-2 cells (p.95-105) were cultured in the control condition, loaded with Fura-2 AM, mounted in an open recording chamber and 350 and 380 nm fluorescence was imaged through epifluorescence microscopy. Baseline fluorescence was measured for 25 min and then for 125 min after the addition of KRH (Control) or sucralose (Suc) at a final concentration of 0.5 or 5 mM (*n* = 4). The graph shows representative kinetics of the Fura-2 AM 350/380 nm fluorescence ratio. **(B)** Changes in Fura-2 AM 350/380 nm fluorescence ratio after sucralose addition. *P < 0.05 compared to baseline. **(C)** Sucralose-induced boost of mitochondrial respiration is independent of the mitochondrial Ca^+2^ uniporter. Caco-2 cells (p.95-105) were cultured in control condition or treated with 0.5 or 5 mM sucralose for 3 h in the presence or absence of 10 μM ruthenium red (RuRed). Then, their oxygen consumption rate was measured by oxygraphy using Clark's electrode (*n* = 6). Baseline, non-ATP-associated (400 nM oligomycin) and uncoupled (12 μM CCCP) respiration conditions were analyzed. *P < 0.05 compared to the baseline of control cells in the same condition of presence or absence of RuRed. All data are shown as mean ± SEM and were analyzed using one-way ANOVA followed by a Holm-Sidak post-test.

Ca^2+^ is a known regulator of mitochondrial bioenergetics. Therefore, we measured cytosolic Ca^2+^ levels by loading Caco-2 cells with Fura-2 AM dye. Once a stable baseline was recorded, cells were exposed to 0.5 or 5 mM sucralose for 2 h. With this experimental design, we aimed to determine whether changes in Ca^2+^ homeostasis precede the mitochondrial effects detected at 3 h. We observed that both sucralose concentrations triggered a sustained increase in cytosolic Ca^2+^ (Figure 4A). Further analysis of the Fura-2 AM signal was performed for each single-cell recording by comparing the fold change to baseline after sucralose treatment (Figure 4B). Because acute increases in mitochondrial Ca^2+^ often stimulate mitochondrial respiration, we hypothesized that it could be the mechanism of the sucralose-induced boost of mitochondrial respiration. Hence, we evaluated the oxygen consumption rate in the presence of RutRed, an inhibitor of MCU1. As shown in Figure 4C, the presence of RuRed did not prevent the sucralose-induced increase in oxygen consumption. On the contrary, sucralose treatment in the presence of RuRed further increased baseline and uncoupled mitochondrial respiration. These results indicate 3 h of sucralose treatment stimulates mitochondrial bioenergetics independently of mitochondrial Ca^2+^ entry. Instead, mitochondrial Ca^2+^ entry acts as a negative regulator of mitochondrial respiration in Caco-2 cells.

## Discussion

Mitochondria function is key in energy homeostasis and its dysfunction is associated with metabolic diseases. Here, we evaluate the acute effect of sucralose on mitochondria function in an intestinal cellular model. In particular, we showed that sucralose increased the reductive power, oxygen consumption and mitochondrial transmembrane potential. However, we do not observe a change in energetic cellular status. To our knowledge, this is the first report showing a direct effect of the sweetener on mitochondrial bioenergetics.

Because sucralose does not enter into cells (19), its metabolic effects are likely initiated at the cell surface and mediated by an intracellular second messenger. In MIN6 cells, sucralose increased cytosolic Ca^2+^ by inducing its mobilization from intracellular stores and also from the extracellular milieu, mediated by the activation of sweet taste receptor T1R2/T1R3 (7). Reportedly, Caco-2 cells express the receptor (12, 20). Therefore, T1R2/T1R3 activation may be the mediator of the sucralose-induced cytosolic Ca^2+^ increase.

The increase in mitochondrial respiration is often a consequence of increased mitochondrial Ca^2+^ uptake (16). A moderate rise in mitochondrial Ca^2+^ leads to the activation of enzymes of the citric acid cycle (TCA) as isocitrate dehydrogenase and oxoglutarate dehydrogenase. Also, Ca^2+^ increases the activity of pyruvate dehydrogenase, which catalyzes the conversion of pyruvate into acetyl-CoA. This a key step in cell mitochondrial metabolism (21). In the same sense, an increase in the activity of TCA encourage OXPHOS to supply reduced equivalents. Consistently, we showed an increase in reductive power in cells treated with sucralose, suggesting an augmented activity in mitochondrial reductases enzymes as succinate dehydrogenase and cytochrome c oxidase. Van Eyk et al. obtained similar results in Caco-2 and HT 29 cells in the presence of 0.1 and 1 mM of sucralose (18) and Shill et al. in Caco-2 cells with 1 mM (12).

The ability of mitochondria to capture Ca^2+^ is mediated for mitochondrial Ca^2+^ channels known as MCU1 (17). We used Ruthenium Red, a chemical inhibitor of this channel. However, it did not block the sucralose effect on mitochondrial respiration, suggesting a mechanism independent of mitochondrial Ca^+2^ entry. In this regard, it is possible that the sucralose-induced increase in cytosolic Ca^+2^ indirectly acts on mitochondrial function through the activity of protein kinases. For example, increased cytosolic Ca^+2^ activates the calcium-sensitive kinase CAMKK2 (also known as CAMKKβ), which augments mitochondrial function, thus linking calcium signaling to the regulation of energy metabolism (22). On the other hand, we do not rule out the participation cAMP, which reportedly increases in MIN6 cells treated with sucralose (7).

Regarding the cellular energy state, although we observed increased oxygen consumption and higher mitochondrial transmembrane potential after sucralose treatment, ATP levels did not change. Our results contrast with Kojima et al. who showed an elevation in ATP levels in response to sucralose (19). Another study by the same research group showed that exposure to 10 mM sucralose induced an increase in ATP levels in the presence of 5.5 mM glucose (23). These different results can be explained because they used a shorter sucralose treatment of 30 min, higher sucralose doses of 10 mM, and a different cell line, β pancreatic cell line MIN6. On the contrary, in *D. melanogaster*, Wang et al. showed that chronic sucralose consumption stimulates the hypothalamic response to fasting through the activation of AMPK, which is a known sensor of AMP/ATP ratio (14). In our case, we did not observe changes in AMPK activation, which agrees with unaltered cell bioenergetics. Altogether, these evidences suggest that sucralose may induce divergent metabolic responses, depending on cell type, exposure time, and organismal context.

On the other hand, a strong positive correlation exists between mitochondrial membrane potential and reactive oxygen species (ROS) (24). At present, it is widely accepted that mitochondria produce more ROS at high membrane potential with inhibition of the ATP synthase (25). Interestingly, studies with sucralose-treated mice suggested increased ROS levels in the liver (11). However, sucralose consumption did not induce oxidative stress in the brain of diabetic rats (26). Recently, it has been reported that sucralose does not increase ROS production in Caco-2 cells, measured with the fluorogenic dye DCFDA at 0.1 mM sucralose (12). Thus, further investigation is required to unravel the relevance of the sucralose-induced increase in mitochondrial transmembrane potential.

A limitation of our study was that we used a cell line as a research model, which excludes the impact of endocrine and neuronal regulation. Nonetheless, high-passage Caco-2 cells (p.95-105) are regarded as a good model of human enterocytes (27). Also, we used a high dose of sucralose (5 mM). However, a lower dose of 0.5 mM is also a potentially reachable dose in the intestine, given the low absorption of the sweetener (12). Finally, we do not measure mitochondrial mass or dynamics associated with mitochondrial function; however, we did measure direct functional parameters, such as baseline and uncoupled respiration, mitochondrial transmembrane potential, and ATP levels.

In synthesis, we showed an acute effect of sucralose on mitochondrial bioenergetics. This may contribute to understand the underlying mechanisms of the metabolic effects of sweetener. Importantly, enterocyte mitochondrial function is relevant for gut permeability (28). Mitochondrial uncoupling increases intestinal permeability, generating local and systemic inflammation (29), which associates with the development of inflammatory bowel diseases such as ulcerative colitis (30). Intriguingly, sucralose consumption exacerbates intestinal inflammatory reactivity in an animal model of Crohn's disease (13), disrupts the intestinal epithelial barrier (12), and epidemiological studies indicate that artificial sweeteners may increase the risk of Crohn's disease (31). Interestingly, recent evidence shows a bidirectional interaction between mitochondria and the gut microbiome (32, 33). Moreover, dysfunctional mitochondria are consistently associated with metabolic diseases such as insulin resistance, diabetes mellitus II and non-alcoholic fatty liver (15). Consistently, studies have reported that sucralose affects glucose homeostasis and liver inflammation (4–6, 15). Nonetheless, these effects are controversial and remain unclear.

## Conclusion

Here, we showed for the first time that sucralose acutely increases mitochondria bioenergetics, characterized by increased oxygen consumption and transmembrane potential, with no changes in ATP production. These effects were independent of mitochondrial Ca^2+^ uptake, which surprisingly, acts as a negative regulator of mitochondrial bioenergetics. This work suggests that sucralose alters enterocyte energy homeostasis, contributing to its effects on organismal metabolism. However, further investigation is required to address its pathophysiological implications, using animal or human models with chronic sucralose consumption.

## Data Availability Statement

The raw data supporting the conclusions of this article will be made available by the authors, without undue reservation.

## Author Contributions

JB, OP, RT, JR, and RB-S: conceptualization. JB, MH, JR, and AM: investigation. OP, RT, and RB-S: project administration. RT and RB-S: supervision. JB: writing original draft. JB, AM, OP, RT, and RB-S: writing—review and editing. All authors contributed to the article and approved the submitted version.

## Conflict of Interest

The authors declare that the research was conducted in the absence of any commercial or financial relationships that could be construed as a potential conflict of interest.
